# Processing strategies to improve the breadmaking potential of whole-grain wheat and non-wheat flours

**DOI:** 10.1007/s44187-022-00012-w

**Published:** 2022-03-02

**Authors:** Tamara Dapčević-Hadnađev, Jelena Tomić, Dubravka Škrobot, Bojana Šarić, Miroslav Hadnađev

**Affiliations:** University of Novi Sad, Institute of Food Technology, Bulevar cara Lazara 1, 21000 Novi Sad, Serbia

**Keywords:** Breadmaking, Whole-grains, Non-wheat cereals, Novel technologies, Cereal processing

## Abstract

Strategies to increase the bio-functionality of staple food, such as bread, by incorporating whole-grain wheat flour or flour from other, non-wheat grains instead of refined wheat flour are often constrained with the lack of their techno-functionality, despite the associated beneficial effect on consumers' health and well-being. Most of the available studies investigating the possibilities to improve technological and sensory quality of bread prepared using whole-grain wheat and non-wheat flours still rely on formulation approaches in which different additives and novel ingredients are used as structuring agents. Less attention has been given to technological approaches which could be applied to induce structural changes on biopolymer level and thus increase the breadmaking potential of whole grains such as: modification of grain and biopolymers structure by germination, flour particle size reduction, dry-heat or hydrothermal treatment, atmospheric cold plasma, high-pressure processing or ultrasound treatment. Strategies to modify processing variables during breadmaking like dough kneading and hydration modification, sourdough fermentation or non-conventional baking techniques application are also poorly exploited for bread preparation from non-wheat grains. In this paper, the challenges and opportunities of abovementioned processing strategies for the development of bread with whole-wheat flours and non-wheat flours from underutilised gluten-containing or gluten-free cereals and pseudocereals will be reviewed throughout the whole breadmaking chain: from grain to bread and from milling to baking. Feasibility of different strategies to increase the technological performance and sensory quality of bread based on whole-grain wheat flours or flours from other, non-wheat grains will be addressed considering both the environmental, safety and nutritive advantages.

## Introduction

Bread, regardless of the type, production process and geographical origin, is traditionally produced from refined common wheat (*Triticum aestivum*) flour. However, in recent years, there has been renewed interest in fortifying or replacing refined wheat flour with whole-grain wheat flour, or flour from gluten-free cereals (rice, maize, sorghum, millet), pseudocereals (amaranth, buckwheat, quinoa) and ancient cereals [1, 2]. This trend is governed with different reasons: from health-conscious and eco-friendly to economically driven.

Unlike refined wheat flour, whole-grain cereals and pseudocereals possess dense nutritional composition and a range of bioactive compounds. Therefore, their consumption contributes to increased intake of micronutrients, dietary fibres, phenolics, etc. Several studies have shown that regular consumption of whole-grain cereals is associated with health benefits such as a lower risk of chronic-degenerative diseases and improved body weight regulation [3]. Additionally, gluten-free cereals are finding an increased demand since coeliac disease or other gluten-associated allergies incidence rates are raising over time [4]. On the other hand, in developing countries, utilization of indigenous grain crops (the case of millet in Africa) is promoted. This contributes to economic development of local agriculture sector through reducing reliance on wheat importation and ensuring food security. Utilization of 'zero km' ingredients and relevance of short food supply chains in increasing the access to healthy and sustainable food has particularly growing attention in crisis situation such as COVID-19 pandemic [5, 6].

Despite their contribution to consumers' well-being, sustainability of cereal cultivation and biodiversity protection, whole-grain alternative cereals exploitation in breadmaking is still being diminished due to the lower technological quality compared to refined wheat. The major challenges encountered in whole-grain or non-wheat cereals incorporation in breadmaking are poor gas retention, low loaf volume, hard and/or crumbling crumb texture, altered colour, short shelf-life of bread. This could be related to dilution or absence of gluten complex responsible for viscoelastic properties of dough and/or water competition effect between fibres and gluten [1, 7]. The abovementioned quality deficiencies are often coupled with the lower consumers' acceptance of the product sensory properties. The most common sensory attributes of whole-grain and non-wheat cereal-based products are nutty odour, pungent flavour, bitter/astringent/sour taste; associated with the presence of phenolic compounds and in particular the condensed tannins which are located in the outermost bran layers [6]. In addition, lipid-rich cereals, such as oat, are susceptible to lipid oxidation which leads to development of the undesired sensory attributes evaluated as musty and earthy odour and bitter and rancid flavour [8]. Generally, altered technological quality (product volume, texture, structure, etc.) and sensory attributes of whole-grain and non-wheat cereal based products represent a limitation in their widespread acceptance.

Different strategies are thus proposed to produce bread from whole-grain and non-wheat cereals with technological and sensory profile comparable to refined wheat bread, while preserving their nutritional value. The most commonly applied strategies are the once involving bread formulation optimization through inclusion of various improvers, such as vital gluten or texturing agents (e.g. hydrocolloids, emulsifiers, enzymes and different food additives) that could act as structure forming agents instead of diluted or absent gluten [9, 10]. In order to contribute to 'clean label' products design as well as its cost-effectiveness, some researches have modified abovementioned compositional approach by replacing food additives with fibre rich raw materials or food processing by-products to overcome the gluten deficiency [11, 12].

However, relatively little research has been conducted on technological approaches for improving breadmaking potential of whole-grain and non-wheat cereals. As noted by Parenti et al. [1] instead of modifying process variables to prepare unrefined wheat flour bread, most of the studies are adopting the same methods as for their counterparts prepared with refined flour.

**Fig. 1:**
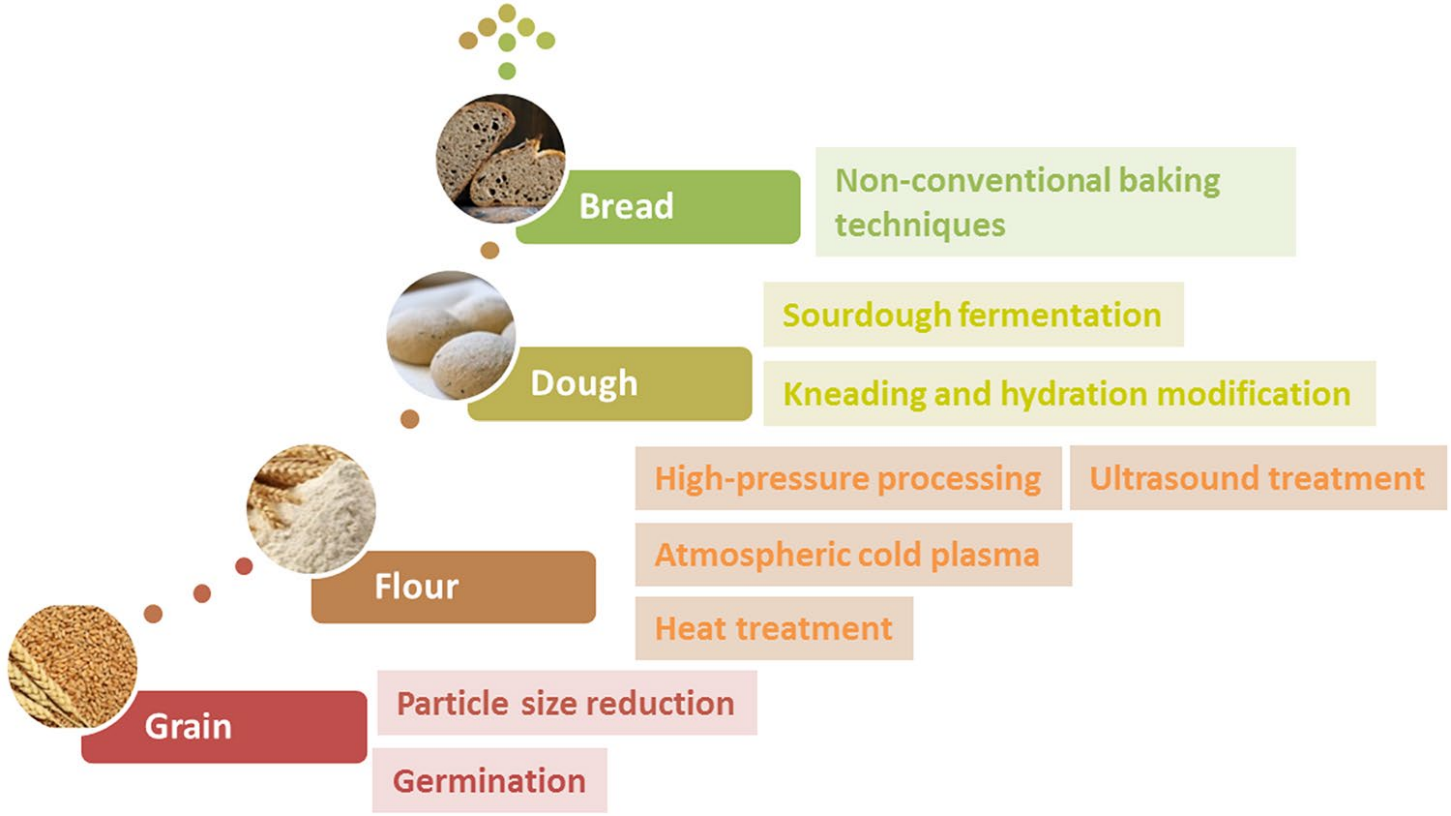
Summary of technological approaches for increased breadmaking potential of whole-grain wheat and non-wheat flours along the whole breadmaking chain

Therefore, the aim of this review is to provide a critical opinion on current and future-looking sustainable technological innovations and strategies utilized to increase the technological performance and sensory quality of bread based on whole-grain and non-wheat cereals. Improvement strategies discussed in this paper encompassed the whole bread production chain (Fig. 1): from raw material (cereal, flour, etc.) to process (milling, kneading, leavening, baking, etc.) modification, considering both the environmental, safety and nutritive advantages related to the use of conventional and emerging technologies and approaches.

## Strategies to modify raw material for breadmaking

### Grain modification approaches

#### Germination

Modification of grain and biopolymers structure by germination is mostly performed to initiate nutrient compositional changes which are associated to health benefits. During the germination process degradation of macromolecules occurs due to increased enzyme activities: (i) starch is hydrolysed by amylolytic enzymes to maltose, glucose, dextrins and oligosaccharides, resulting in its higher digestibility [13–15]; (ii) storage proteins are degraded by endopeptidases produced from the aleurone layer and scutellum thus releasing peptides and free amino acids [15–18]; (iii) the ratio of soluble to insoluble dietary fibre increases especially when long germination times are applied [17, 20]; (iv) a phytate (antinutrient present in cereals) content decreases as a result of increased phytase activity thus releasing chelated cations leading to increased bioavailability of phosphorus and minerals such as Zn^2+^, Fe^2+/3+^, Ca^2+^, Mg^2+^, Mn^2+^ and Cu^2+^ [13]. Moreover, germination process results in the increase in free fraction of phenolic acids due to decrease in the bound one contributing to increased antioxidant activity [13, 15, 18, 19]. Germination is also a strategy to produce important metabolites such as γ-aminobutyric acid (GABA) [14, 18], recommended to prevent neurological disorders [21].

Although increase in enzymatic activity produced by germination has mostly a detrimental effect on the breadmaking potential of cereals, with proper adjustment of the germination parameters it can be a promising tool to improve both the nutritional and technological properties of cereal-based food. In general, germination leads to softer and more fragile grain as a consequence of enzyme action which results in lower damaged starch content upon milling [22]. This, along with partial protein hydrolysis and decrease in insoluble fibre content, contribute to lower water absorption of flour from germinated wheat [17]. The germination also affects dough rheological properties in the following directions: (i) weakening of the gluten ability to form viscoelastic network due to decrease in the level of high-molecular-weight glutenin macropolymers which reflects in reduction of the tenacity, an increase of the extensibility of dough, and (ii) reduction of starch gelatinization and retrogradation ability as a result of hydrolysis [14, 23, 24].

However, shorter germination times, low substitution levels or addition of some improvers (vital wheat gluten) to germinated wheat flour could increase technological performance of whole-grain cereals [1, 17, 25]. Activation of slight amount of α-amylase will increase starch transformation to fermentable sugars thus promoting yeast fermentation, carbon dioxide production and increase in dough height during fermentation [26, 27], which, along with increased dough extensibility, will contribute to gas cell expansion leading to bread loaves of higher specific volumes as evident from the study of Baranzelli et al. [14], Johnston et al. [28], Cardone et al. [29] and Bhinder et al. [18] (Table 1).

**Table 1:** Impact of germination on the quality of leavened bakery products prepared from whole-grain wheat flour or flour from non-wheat grains

Germination conditions	Cereal used	Effect in leavened bakery product	Reference
t = 24, 48 and 72 h; 80% relative humidity, T = 15 and 20 °C, at intervals of 12 h at each temperature	100% germinated white wheat flour	Wet gluten content—increased for samples germinated 24 and 48 h, decreased for sample 72 h Dough extensibility—increased Water absorption—decreased Dough stability—decreased Bread specific volume—increased up to 48 h, at 72 h slight decrease Crumb firmness—increased Bread lightness (L^∗^)—decreased at 24 and 48 h, increased to control value at 72 h	[14]
t = 24 h, T = 21 °C, excess of water, Falling Number reduction from 350 to 200 s	100% germinated whole-wheat flour	Dough mix time—decreased Loaf volume—increased Consumer preference of whole-grain bread—increased	[28]
t = 48 h, T = 20 °C, 90% relative humidity	100% germinated whole-wheat flour	Dough extensibility—increased Water absorption—decreased Dough development time—decreased Dough stability—decreased Bread specific volume—increased Crumb softness—increased	[29]
t = 24, 48, 72 and 96 h, T = 24 °C, in the dark conditions, intermittently sprayed with water after 8 h	20% and 40% germinated tartary buckwheat and non-germinated rice flour	Specific volume—increased for samples germinated 24 and 48 h; 72 and 96 h detrimental effect Firmness—decreased for samples germinated 24 and 48 h Bread lightness (L^∗^)—decreased	[18]

In addition, optimized α -amylase activity can improve the bread shelf-life and sensory attributes [17]. It was shown that due to restricted starch retrogradation, germination improved crumb softness for 200% after 24 h of storage even when whole-wheat flour was used [29]. Controlled germination can also yield a product of enhanced starch digestibility [15] and reduced glycaemic index [18]. Moreover, germinated whole-wheat breads had improved sensory attributes in comparison to their unsprouted counterparts thanks to their diminished bitterness and graininess, increased sweetness and moistness [25, 28]. Breads with germinated wheat flour are also perceived as the ones with dark crust due to the presence of higher contents of reducing sugars that, combined with free amino acids, favoured the occurrence of a Maillard reaction [14].

### Flour modification approaches

#### Particle size reduction (micronization)

Flour particle size can significantly alter bread functionality and technological quality. If a micronization, such as jet milling, is applied to produce fine wheat flour with extremely low particle size, flour with increased digestible starch content is obtained [30]. When used in breadmaking, jet milled flour slightly decreased bread glycaemic index.

However, it seems that pulverization of flour is not promising technology concerning bread technological quality since whole-grain wheat jet milled breads (flour volume median diameter = 17–53 μm) were characterized with reduced specific volume and moisture content and increased crumb hardness in comparison to breads with flour having volume median diameter of 84 μm [30]. The same relationship between flour mean particle size and technological performance was obtained for gluten-free flours. The flours having coarser particle size are the most suitable for making gluten-free maize bread. According to de la Hera et al. [31], the coarser maize flours (> 150 µm) resulted in breads with higher specific volume and lower crumb firmness than the ones with finer flour (< 106 µm), due to the higher availability of dough to retain the gas produced during fermentation. Concerning rice flour incorporation in breadmaking, de la Hera et al. [32] concluded that the coarse fraction combined with a high dough hydration was the most suitable combination for developing rice bread when considering the bread volume and crumb texture.

#### Heat treatment

Different flour heat treatments such as dry-heat treatment or hydrothermal treatments (below or above starch gelatinization temperature) are being increasingly applied to improve the functionality of alternative cereals flour. It was shown that dry-heat treated sorghum flour produced breads with increased specific volume and more cells per slice area. This was ascribed to increased viscosity of sorghum flour dough as a consequence of starch granule swelling due to heat induced partial gelatinization as well as denaturation of both proteins and enzymes [33]. In addition, protein denaturation and the partial gelatinization of starch granules, led to an increase in gas retention capacity and dough expansion, which all contributed to improvements in structure, strength and volume of dry-heated sorghum containing bread [34]. Since sorghum-based products are characterized with pungent off-notes, dry heat treatment can also be employed to improve sorghum bread sensory properties [35]. Dry heating was also promising in upgrading the quality of substandard flour for bread-making applications [36]. Mann et al. [37] have shown that heat treatment of flour causes the formation of gluten and starch aggregates and modifies interactions between gluten and starch. The effects were more pronounced in heat-treated flours with increased moisture content where higher mobility of the molecules is enabled.

It was also revealed that gluten-free flours (maize or rice) blanching results in doughs with higher consistency, adhesiveness, springiness and stickiness due to the partial gelatinisation of the starch, which further led to improved bread quality [38, 39].

When flour/starch heating is carried out in the presence of water without fostering a complete starch gelatinization, as it is the case with annealing (treatments in excess or at intermediate water contents below the gelatinisation temperature) and heat-moisture treatment (exposure of starch to higher temperatures at very restricted moisture content), increase in the starch gelatinization temperature, water binding capacity and granule susceptibility to enzyme hydrolysis occurs [40, 41]. These structural changes improve the volume of breads and their quality, since restricted hydrothermal treatments increase starch emulsifying ability and delay gelatinization which enhance air incorporation in doughs and prolong the period of loaf expansion [40].

It was shown that application of hydrothermally treated rice and maize flour to manufacture rice and maize semolina-based breads increased the specific volume and decreased the hardness and chewiness of the gluten-free breads, due to higher initial viscosity imparted by treated flours enabling the entrapment of air bubbles in the dough [42].

When hydrothermal treatments are performed above gelatinization temperature starch granules are irreversibly losing their integrity, a process known as pre-gelatinization [40]. Parenti et al. [43] reported an increase in the water absorption capacity, improved alveograph parameters, as well as bread volume, crumb softness and shelf life when pre-gelatinized brown flour (flour having approx. 85% extraction yield, maximum ash content of 0.95 g/100, heated at 1:4 flour to water ratio at 85 °C) was used. Jalali et al. [44] used microwave-induced pre-gelatinization of maize flour to produce gluten-free pan bread. The authors observed structural expansion and more swelling of the pre-gelatinized maize flour as compared to non-treated one, which consequently resulted in increased firmness of dough, decreased firmness of bread, increased bread crumb moisture, porosity, loaf specific volume and the overall acceptability.

If pre-gelatinization is achieved with the aid of extrusion cooking (flour/starch exposure to high temperatures and mechanical shearing with enough amount of water) besides amylose and amylopectin leaching from disrupter starch granule, breakage of the amylose and amylopectin chains, denaturation of proteins, enzyme (in)activation and Maillard reactions occurred [40]. Extrusion cooked flour behaves as thickening agent [45], which is considered as a more 'natural approach' to the use of hydrocolloids as improvers. Substitution of native rice flour by extruded rice flour improved bread volume and crumb structure, decreased initial hardness and delayed bread staling in gluten-free bread [46].

#### Atmospheric cold plasma

Atmospheric cold plasma (ACP) is a non-thermal processing technology that so far was applied at different stages of the cereal processing chain for a range of applications including improved germination, microbial decontamination, toxin degradation and biopolymer structural changes for improved functionality [47]. The mode of action results from plasma generated reactive species (reactive oxygen and nitrogen species), radicals and UV light [48]. It was revealed that reactive oxygen species generated during wheat flour cold plasma treatment influenced protein oxidation, promoted disulfide bond formation between glutenin proteins, that improved dough strength; led to starch depolymerization and decrease in its crystallinity. These biopolymer structural changes reflected in the increase in bread specific volume, enhancement of its appearance and porosity structure, as well as increase in bread crumb whiteness [49–51].

However, most of the studies investigating plasma-induced changes in grain/flour/dough structure are based on breadmaking potential of refined wheat flour, biopolymer changes in whole grain wheat or the safety aspects of plasma application for alternative grains decontamination. The studies concerning plasma application to enhance breadmaking performance of whole-grain or non-wheat cereals are scarce. Since some preliminary studies have shown that ACP treatment is effective just in increasing breadmaking potential of weak flours [52], some future studies should be conducted for better exploitation of ACP in whole-grain of gluten-free cereals modification. Moreover, combination of different technologies such as plasma-activated water and heat moisture treatment can also offer novel possibilities in alternative grains utilization in breadmaking [53].

### Dough modification approaches

#### High-pressure processing

High-pressure processing (HPP) represents novel processing technology which is mainly used for non-thermal treatment for fruit juices preservation [54]. Generally, in high-pressure processing, food is subjected to high pressures (usually above 200 MPa, without high temperature treatment) causing structural and textural changes besides microbial inactivation. These changes are mainly influenced by starch gelatinization and polymerization of proteins [55]. Therefore, this technology can be effectively employed for protein and starch functional properties modification [56]. Moreover, Kieffer et al. [57] revealed that high pressure treatment promotes protein network formation. Most of the papers using HPP in cereal technology is mainly focused on gluten-free raw material treatment due to poor technological properties of these materials i.e. the lack of protein network formation, poor gas retention properties, poor volume, acceptability etc. Generally, it was determined that HPP treatment resulted in starch gelatinization and protein polymerization induced by reaction of thiol-disulfide interchange. Consequently, the dough became more viscoelastic, showed better workability, increased water absorption capacity and had better gas retention properties which resulted in increased volume and improved texture of the final product [58, 59]. Moreover, the obtained bakery products had improved shelf life [60] and slower hardening kinetics in comparison to control samples, due to starch gelatinization that occurred in this process. However, according to Vallons et al. [61] the increase in the addition of pressure treated flour over 10% resulted in lower specific volume and poorer final product quality.

#### Ultrasound treatment

Ultrasound treatment, as a non-thermal processing tool, has been intensively utilized for microbial and enzyme inactivation, bioactive component extraction and food components modification for increased functionality [62]. However, application of ultrasound to alter flour functionality and thus improve its breadmaking potential is quite scarce.

While it was shown that ultrasound modulation of flour functionality depends on the treatment time [62, 63], there are opposite conclusions concerning the effect of the flour dispersion concentration. According to Vela et al. [63], effect of ultrasound treatment is independent on the concentration of the treated flour dispersion up to 30%, and in all the treated dispersions (5–30%) particle size of the rice flour was reduced. On the contrary, ultrasound treatment of buckwheat grains caused particles agglomeration in concentrated dispersions (1:5 and 1:2.5 solid:liquid ratio), while higher dilution (1:10) increased smaller particle size fractions [64].

In general, ultrasound treatment of whole-grain flour significantly increases water solubility, water absorption and swelling power of quinoa, buckwheat and rice flour [62–64]. It also influences starch crystallinity as recorded in the alterations of the flour thermal properties such as reduction of gelatinization enthalpy, increase in pasting temperature and gel strength [63], as well as in an increase in the in vitro starch digestibility [62]. However, effects on the flour pasting properties were found to be dependent on treatment time [62] and dispersion concentration [64], where lower treatment times [62] and medium concentrations [64] led to increase in peak viscosity, breakdown, and setback values.

Jalali et al. [44] have shown that ultrasound treatment of dough decreased the firmness of maize flour dough and bread, while increasing gluten-free bread specific volume, porosity, and the overall acceptability score. The observed improvement in bread technological, visual, and sensory properties was increased when combination of pre-gelatinization and ultrasound treatment of maize flour was applied [44].

## Strategies to modify processing variables of the breadmaking phases

### Dough kneading and hydration modification

Flour transformation to dough is performed by hydration and mixing operations, where different processing variables can be modified in order to achieve optimum dough and bread quality. Appropriate water content and temperature ensure optimal dough rheology and consistency, avoiding undesired softening or hardening. Proper choice of mixing speed and temperature will avoid dough warming and excessive weakening, while kneading time management prevents both over- and under-mixing and allows dough aeration and its capacity to retain gases [5].

Water content influences dough quality in the following manner: adding too much water during kneading generates soft and sticky dough, while dough with water content below the optimal water absorption of the flour will be harder to knead [5]. Increase in total water content in dough from ancient grain flours increases dough extensibility, while it decreases dough tenacity and vice versa [65]. In the case of gluten-free ingredients, such as rice flour and hydroxypropyl methyl cellulose (HPMC), low hydrated doughs had low ability to retain gas released during proofing, unlike high hydrated doughs which endure longer fermentation time resulting in improved specific volume [66]. Therefore, different strategies are applied in order to increase water absorption and thus improve gluten-free bread quality. Due to the absence of gluten in gluten-free ingredients, increased water absorption is achieved through fibres/hydrocolloids addition or enzymatic or extrusion treatments to modify amount of water which will be untaken by starch in the early phases of breadmaking [67, 68].

Gomez et al. [66] have also reported that low mixing speed and long mixing time led to gluten-free breads with higher specific volumes and softer texture.

### Sourdough fermentation

Although being an ancient biotechnology, sourdough fermentation has gained renewed interest as a tool for better exploitation of non-wheat cereals in breadmaking [69]. Sourdough can be described as a mixture of flour and water fermented by lactic acid bacteria (LAB) or LAB in combination with yeasts, either spontaneous or inoculated [70]. The positive effects of sourdough application in breadmaking are associated with the metabolic activities of the LAB and yeasts, such as acidification, production of exopolysaccharides, proteolytic, amylolytic and phytase activity, and production of volatile and antimicrobial substances [71].

Beside the fact that sourdough fermentation contributes to enhanced nutritional properties of bread (higher free amino acids concentrations, soluble fibre, γ-aminobutyric acid, total phenols and antioxidant activities) and phytic acid reduction, leading to increased mineral, protein and free amino acids bioavailability; it has significant impact on bread techno-functionality [6, 72].

Taking advantage of LAB ability to produce certain polymers and modify the main structure-building components of flour such as starch, arabinoxylans and proteins, sourdough fermentation was used to improve dough and bread technological properties such as loaf volume, water absorption of the dough, dough rheology and machinability [73]. Certain LAB strains produce exopolysaccharides that due to their water-binding ability act as hydrocolloids or gums, and could be considered as gluten mimetics in gluten-free products [74] in order to improve product texture. In gluten containing flours, organic acids produced by LAB enhance the solubility of the glutenin fraction and improve the swelling power of the gluten, which increase gas retention during fermentation [73]. Gluten complex structural changes are associated with dough acidification which may also activate some endogenous flour enzymes such as proteases that can hydrolyse gluten under appropriate fermentation conditions and bacteria selection. Gobbetti et al. [75] suggested that degradation of prolamins of wheat and rye during fermentation by selected sourdough lactic acid bacteria can represent a possibility to use these cereals in the gluten-free diet.

On the contrary, reports on the fate of starch during sourdough fermentation are contradictory. In the case of the wholegrain wheat flour, sourdough fermented bread exhibited higher resistant starch content and lower glycaemic response than the corresponding products leavened with *S. cerevisiae* [76]. However, sourdough with a commercial starter added to a gluten-free formulation decreased the glycaemic response in vivo less effective than in wheat sourdough bread. This was explained with lower concentrations of organic acids in gluten-free than in wheat sourdough. In sourdough wheat breads pH decrease upon formation of organic acids led to inhibition of α-amylase and consequently, a decrease in starch hydrolysis. On the contrary, the pH in gluten-free sourdoughs might still be sufficient for α-amylase to proceed with degradation of starch and increase in starch hydrolysis degree [77].

The effect of sourdough fermentation on techno-functionality of bread prepared with alternative cereals is summarized in Table 2. As it can be seen from Table 2, the effect of sourdough addition on bread technological performance largely depends on sourdough type, LAB strain and presence of *Saccharomyces cerevisiae.*

**Table 2:** Effect of sourdough fermentation on the quality of bread prepared from whole-grain wheat flour or flour from non-wheat grains

Fermentation type/strain	Cereal in breadmaking	Effect in bread	Reference
Type-1 (spontaneous fermentation)	Whole wheat flour bread	Lower specific volume/higher hardness compared to control with dry yeast	[78]
Type-2 (*Lactobacillus brevis ELB99, Lactiplantibacillus plantarum ELB75, and Saccharomyces cerevisiae TGM55*)			
*L. brevis*	Pearl millet-based bread	Sourdough breads retained their moisture better than conventional loaves; suppressed the development of mould for a longer period; and were more palatable than conventional or chemically acidified ones	[79]
*Lactobacillus plantarum, Lb. brevis* or *Leuconostoc mesenteroides* mixed with yeast *Candida humili*	Wholemeal wheat flour bread	*Lactobacillus plantarum* sourdough addition most efficiently retarded firming rate and improved specific volume in comparison with partially baked frozen bread without sourdough	[80]
Inoculated with multi-strain starter culture (LAB and yeasts) and fermented with the back-slopping technique	Hull-less barley bread	Lower specific volume, harder and denser crumb in comparison to wheat bread, but comparable overall acceptability scores	[81]
Commercial starter cultures (Lesaffre, Wołczyn, Poland)	Gluten-free amaranth bread	Application of fresh and freeze-dried amaranth sourdough led to increased bread volume, decreased crumb hardness and larger pores on the crumb (10% sourdough addition was sensory more preferred than 20% addition)	[82]
*Lactobacillus amylovorus* DSM19280 and *L. amylovorus* DSM20531^T^ and yeast	Gluten-free quinoa bread	Softer crumb, higher specific loaf volume in comparison to non-acidified bread	[83]
Gluten-free inoculum from sourdough water	Gluten-free teff bread	The bread enriched with fermented teff had higher specific volume, softer bread crumb and a lower staling rate with respect to a bread enriched with non-fermented teff flour	[84]
*Pediococcus pentosaceus* SA8, *Weissella confusa* SD8, *P. pentosaceus* LD7 and *Saccharomyces cerevisiae* YC1	Sorghum flour bread	Sorghum sourdough breads exhibited increased crumb hardness and similar bread specific volume to control bread with YC1	[85]
*Pediococcus pentosaceus* (strain MB33)*, Weissella cibaria* (strain CM32)*,* and *Saccharomyces cerevisiae* strain	Spelt flour bread	Decrease in bread specific volume and crumb texture compared to bread leavened with *S. cerevisiae* only	[86]

Besides bread technological quality, organic acids together with other LAB metabolites (e.g. CO_2_, ethanol, diacetyl, hydrogen peroxide, fatty acids, reuterin, fungicin, etc.) also contribute to bread preservation thus prolonging its shelf life [54]. Sourdough was also successfully applied in a sugar reduced bakery product, owning to sourdough bacteria ability to produce polyols [87]. Because of the synthesis of flavouring amino acids during fermentation, the sourdough efficiently masks salt reduction in bakery products without affecting taste and other quality parameters [88].

### Non-conventional baking techniques

Another interesting approach to improve the breadmaking potential of alternative cereals is to apply a non-conventional baking technique such as vacuum, microwave, infrared, jet-impingement, ohmic or a combination of them (hybrid heating).

In comparison to conventional, partial-vacuum baking of gluten-free bread did not have significant impact on bread volume and texture; however, it resulted in product which became stale more slowly than the control [89].

Microwave and infrared baking are considered as time- and cost-efficient processes. Although microwave and microwave-assisted hot air baking increase gluten-free bread crumb hardness and result in pale bread crust compared with the hot air baking, it was shown that these techniques can reduce the digestibility of starch and glycaemic index of the bread and increase loaf volume [90].

Application of single infrared radiation (halogen lamp as NIR source) results mostly in products of inferior quality, due to the high rate of heating which influence sudden and thick crust formation and the prevention of the product expansion thus leading to lower specific volume and higher firmness values than conventional baking [91, 92]. However, in the study of Shyu et al. [93] breads baked by IR had comparable quality in terms volume, water activity, staling rate, or sensory scores with conventionally baked ones.

Another novel baking technique, jet impinging, based on forced convection heating, increases the heat transfer efficiency during the baking process [94], but results in the formation of a thick crust as compared with infrared radiation and heating in a conventional household oven [95].

Ohmic heating is an innovative technology in which an alternating electrical current is passed through a material, generating heat by dissipation of the electrical energy due to material's own electrical resistance, allowing rapid and uniform heat distribution [54].

Bender et al. [96] have shown that gluten-free breads could benefit from the uniform rapid heating during processing, as these breads exhibit higher loaf volume, finer pore structure, reduced starch digestibility and higher resistant starch content compared to conventionally baked breads. Namely, rapid heating stabilizes the crumb structure at an early stage of baking before CO_2_ is released during heating enabling bread expansion.

In order to increase the potential of non-conventional baking techniques while minimizing the disadvantages a combination of them (hybrid heating) can be applied. Combination of infrared lamps and electric heating coils enables 28% reduction in baking time, while resulting in breads comparable with breads baked in conventional electrical heating in terms of crumb firmness, volume, moisture content and colour [97]. However, there are limited studies applying hybrid heating to produce alternative cereals bread. Demirkesen et al. [98] compared the quality of the gluten-free breads based on the blends of tigernut flour/rice flour baked in conventional ovens and infrared–microwave combination. They observed higher loaf volume and crumb firmness and less gelatinized starch of IR- microwave baked breads. Moreover, staling of gluten-free breads was not affected by both baking methods [99].

**Fig. 2:**
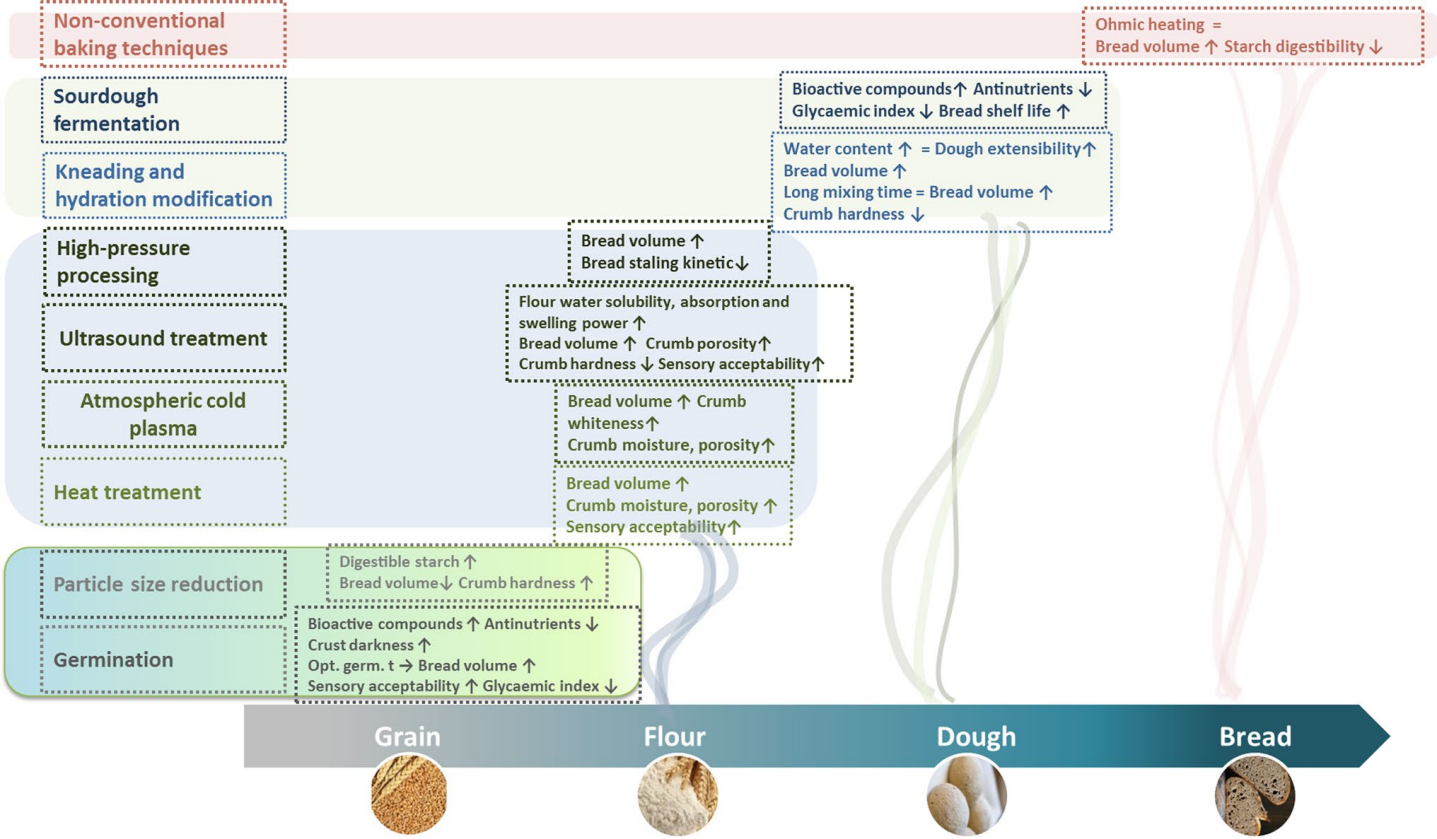
Impact of different technological approaches on breadmaking potential of whole-grain wheat and non-wheat flours

Impact of abovementioned processing strategies on breadmaking potential of whole-grain wheat and non-wheat flours is summarized in Fig. 2.

## Conclusions and future trends

This review has highlighted that different technological strategies can be used to increase techno-functionality of whole-grain wheat and non-wheat flours and sensory properties of final product—bread. They are mostly performed with the aim to alter biopolymer structure and thus increase its functionality and encompass the ones used to provoke starch pre-gelatinization (high-pressure processing, flour heat treatment), reduce starch retrogradation (germination, extrusion cooking, non-conventional baking techniques), induce gluten strengthening through oxidation (atmospheric cold plasma) or gluten hydrolysis (grain germination, sourdough fermentation). It was elucidated that despite the opportunities offered by different conventional and emerging technologies and approaches, the gaps between technological and nutritional strategies for improving breadmaking potential of whole-grains still exist, especially when other, non-wheat grains are used. Namely, effectiveness of reviewed technological approaches largely depends on initial flour composition and quality. Therefore, further investigations are needed, particularly with respect to the ones including combined technologies (atmospheric pressure plasma/thermal treatment; pre-gelatinization/ultrasound; hybrid heating, etc.) to further increase technological and sensory quality of bread from whole-grain non-wheat cereals while preserving health beneficial properties.

## Data Availability

The data that support the findings of this study are available from the corresponding author upon reasonable request.
